# Müller glial cells located in the peripheral retina are more susceptible to high pressure: implications for glaucoma

**DOI:** 10.1186/s13578-023-01186-1

**Published:** 2024-01-05

**Authors:** Xandra Pereiro, Noelia Ruzafa, Mikel Azkargorta, Félix Elortza, Arantxa Acera, António Francisco Ambrósio, Ana Raquel Santiago, Elena Vecino

**Affiliations:** 1 Experimental Ophthalmo-Biology Group, Department of Cell Biology and Histology, University of the Basque Country UPV/EHU, 48940 Leioa, Spain; 2 Proteomics Platform, CIC bioGUNE, Basque Research and Technology Alliance (BRTA), CIBERehdProteoRed-ISCIII, Bizkaia Science and Technology Park, 48160 Derio, Spain; 3 Faculty of Medicine, Coimbra Institute for Clinical and Biomedical Research (iCBR), University of Coimbra, Coimbra, Portugal; 4 Center for Innovative Biomedicine and Biotechnology (CIBB), University of Coimbra, Coimbra, Portugal; 5 Clinical Academic Center of Coimbra (CACC), Coimbra, Portugal; 6 Association for Innovation and Biomedical Research on Light and Image (AIBILI), Coimbra, Portugal

**Keywords:** Müller glia, Retinal ganglion cells, Glaucoma, Retina, Neurodegeneration

## Abstract

**Background:**

Glaucoma, a progressive neurodegenerative disease, is a leading cause of irreversible vision loss worldwide. This study aims to elucidate the critical role of Müller glia (MG) in the context of retinal ganglion cell (RGC) death, particularly focusing on the influence of peripheral MG sensitivity to high pressure (HP).

**Methods:**

Co-cultures of porcine RGCs with MG were isolated from both the central and peripheral regions of pig retinas and subjected to both normal and HP conditions. Mass spectrometry analysis of the MG-conditioned medium was conducted to identify the proteins released by MG under all conditions.

**Results:**

Peripheral MG were found to secrete a higher quantity of neuroprotective factors, effectively promoting RGC survival under normal physiological conditions. However, under HP conditions, co-cultures with peripheral MG exhibited impaired RGC survival. Moreover, under HP conditions, peripheral MG significantly upregulated the secretion of proteins associated with apoptosis, oxidative stress, and inflammation.

**Conclusions:**

This study provides robust evidence suggesting the involvement of MG in RGC death in glaucoma, thus paving the way for future therapeutic investigations.

**Supplementary Information:**

The online version contains supplementary material available at 10.1186/s13578-023-01186-1.

## Background

Retinal ganglion cells (RGCs) are the neurons that transmit visual information from the eye to the brain and thus, their survival is critical for vision. These neurons are very sensitive to insults, and damage to RGC axons in the optic nerve may lead to rapid RGC death in acute diseases like ischemic optic neuropathy or optic neuritis, or in chronic diseases like glaucoma [1]. Different RGC subtypes respond distinctly to stimuli and insults, and not as a single entity, such that their type-specific vulnerability has been studied extensively. As such, early functional alterations to certain subtypes of RGCs have been proposed [2–5]. In glaucoma, there is a greater loss of large RGCs in the peripheral retina, which also occurs in animal models of glaucoma that resemble the features described in glaucoma patients [6, 7].

Müller Glia (MG) are the principal macroglial cells in the retina. They are radially oriented and span the entire thickness of the retina. These cells are responsible for the homeostatic and metabolic support of retinal neurons, making them essential for neuronal survival [8]. Reactive MG can maintain extracellular homeostasis during retinal insult or damage, thereby protecting retinal neurons [9]. These neuroprotective effects of MG may be mediated by diverse mechanisms, enhancing physiological functions like glutamate or potassium uptake. Moreover, neuronal survival can be further preserved through the release of neuroprotective factors by MG [10–12].

MG also represent a heterogeneous population, in which some cells participate more closely in retinal repair than others. Although little is known about the heterogeneity of MG, only one subset of MG express the Chx10 transcription factor [13], while 30% of MG express class II MHC antigen in vitro, suggesting that they might be involved in immune reactions [14]. In terms of their distribution in the retina, MG morphology varies in relation to retinal topography [15]. The central retina is thicker than the periphery, with a greater density of neurons and MG. Moreover, MG from the central retinal are longer and thinner than those in the periphery, and they have a smaller volume but a higher surface to volume ratio [16]. In the chick retina, the region in which proliferating MG accumulate in response to retinal damage becomes mainly confined to the periphery [17]. Two types of MG were distinguished in the chick retina, referred to as type I cells with numerous thin processes, and type II cells with fewer and thicker processes [18, 19]. When characterized further, the type I cells were seen to be in the majority across the retina, whereas type II MG were mainly found in the peripheral retina [20]. Moreover, while mammalian MG lose the capacity to proliferate and regenerate, unlike other vertebrates as fish, peripheral MG express proteins characteristic of stem cells after damage, such as CD44 [21] and the neural progenitor marker, nestin [22]. In the human retina, the microenvironment of the central retina not only has specific characteristics with respect to the periphery but also, it is more susceptible to certain diseases [23]. Phosphoglycerate dehydrogenase, the rate-limiting enzyme in serine synthesis, is expressed more in MG localized in the macula than in peripheral MG, suggesting higher susceptibility of macular cells to oxidative stress than peripheral cells [24]. Macular MG also express more aquaporin-4 than peripheral MG [25]. Furthermore, in a mouse model of laser-induced glaucoma, the strongest activation of macroglial cells was in the intermediate and peripheral retina relative to the central retina [26].

MG isolated from the central or peripheral retina consistently respond in a different manner to the conditions to which they are exposed, indicating molecular differences between the MG from these two different locations [24]. The differences in protein expression among MG at distinct retinal locations may indicate that they fulfill different functions, although this still needs to be extensively studied.

MG are considered the principal retinal sensor that can respond promptly to mechanical changes [27]. The heterogeneity of MGs and their ability to sense pressure could explain the differences in susceptibility to death of peripheral RGCs in glaucoma. As such, MGs may play an important role and could offer insights into the potential causes of the onset of glaucomatous damage. It is important to know if the MG located in the retinal area where the RGCs die first in glaucoma behave differently to those in other retinal areas and if they are also more vulnerable to changes in intraocular pressure (IOP). To address this issue and based on our experience in handling MG and RGCs in culture, we first studied, using co-cultures, the interaction of MG isolated from the peripheral and central retina with RGCs, focusing on the survival of these cells. Subsequently, we examined the effect of HP on these co-cultures using central and peripheral MG, focusing on the susceptibility to death of both MG and RGC. Finally, we compared the proteome of MG conditioned medium in the experimental conditions tested, exposure or not to HP, to identify proteins secreted by MG from the central and peripheral retina. Thus, here we examined the possible different protective effect of MG located in the central or peripheral retina on RGCs, identifying proteins that may be involved in these events. The data obtained led us to suggest that peripheral MG may be involved in the initiation of glaucomatous damage.

## Methods

### Animals

This study was carried out in strict accordance with the Guidelines for the Care and Use of Laboratory Animals. All the experimental protocols complied with the European (2010/63/UE) and Spanish (RD53/2013) regulations for the protection of experimental animals, and they were approved by the Ethical Committee for Animal Welfare at the University of Basque country. All animal experimentation adhered to the ARVO Statement for the Use of Animals in Ophthalmic and Vision Research.

For MG cultures, adult porcine eyes were obtained from a local abattoirs and transported to the laboratory in cold CO2-independent medium (Life Technologies, Carlsbad, CA, USA) containing 0.1% gentamicin (Life Technologies, Carlsbad, CA, USA). For pure RGC cultures, eyes were obtained from adult female Sprague–Dawley rats (200–250 g) housed on a 12 h light–dark cycle with ad libitum access to food and water, and they were sacrificed humanely by exposure to CO_2_.

### Porcine retinal Müller glia cultures

MG cultures were prepared as described previously [28]. First, dissecting adult porcine eyes within 2 h of enucleation. Briefly, the major blood vessels were removed and the retina was washed in CO_2_-independent medium. The retinas were dissected out and two different areas of the retina were obtained with an 8 mm diameter dissecting trephine (Biomedical Research Instruments, Silver Spring, MD, USA): central and peripheral. The retinal tissue was dissociated for 30 min at 37 °C in 0.2% activated papain (Worthington, Lakewood, NJ, USA) with 10% DNAse I (Worthington, Lakewood, NJ, USA). Enzyme activity was stopped by the addition of MG medium (see below) and DNase I. The tissue was then disaggregated by gentle trituration using pipette tips of decreasing diameter.

Purified MG were cultured in DMEM (Life Technologies, Carlsbad, CA, USA) with 10% fetal bovine serum (FBS: Life Technologies, Carlsbad, CA, USA). MG and RGCs were co-cultured in Neurobasal A medium (NBA: Life Technologies, Carlsbad, CA, USA) supplemented with 10% FBS and 2% B27 (Life Technologies, Carlsbad, CA, USA). In addition, 1% l-glutamine (2 mM: Life Technologies, Carlsbad, CA, USA) and 0.1% gentamicin (50 mg/mL: Life Technologies, Carlsbad, CA, USA) were added to the culture media of purified MG and to co-cultures.

Dissociated cells were pelleted by centrifugation (1200 rpm, 5 min), resuspended in MG medium and plated on poly-l-lysine (100 μg/mL: Sigma-Aldrich, St. Louis, MO, USA) and laminin (10 μg/mL: Sigma-Aldrich, St. Louis, MO, USA) coated 13 mm diameter glass coverslips in 24-well plates. The cells were maintained in a humidified incubator at 37 °C in an atmosphere of 5% CO_2_. Cells in suspension were removed after 24 h by changing the medium. For maintenance, half of the medium was replaced every 2–3 days.

Conditioned medium (CM) from MG cultures was collected when the cultures reached confluence at day 7. First carefully washing the wells three times with NBA medium, and subsequently adding fresh NBA medium to each well for 3 h before the medium was changed to eliminate the rest of the FBS and B27. Fresh NBA medium was added and conditioned over 2 days before it was collected sterilized by passing through a 0.22 μm filter and frozen in aliquots at − 20 °C. Finally, the MG were fixed for 10 min with methanol at − 20 °C. At least three replicates of each culture were set-up and the procedure was performed at least in triplicate.

### Purified RGC cultures

RGC cultures were prepared as described previously [29] from a mixed suspension of retinal cells obtained from pig or rat retinas. Pig RGCs were obtained from the center and periphery of the retina, and rat RGCs were obtained from the entire retina. Retinal tissue was dissociated enzymatically using the Papain Dissociation Kit (Worthington Biochemical, Lakewood, NJ, USA), following the manufacturer’s instructions, namely digesting the tissue for 90 min at 37 °C in 0.2% activated papain with 10% DNAse I. After gentle trituration using pipette tips of decreasing diameter to disaggregate the tissue, and after using a density gradient, the purified RGCs were plated on 13 mm poly-l-lysine (100 μg/mL, Sigma-Aldrich, St. Louis, MO, USA) and laminin (10 μg/mL: Sigma-Aldrich, St. Louis, MO, USA) coated glass coverslips in 24‐well plates. The pig and rat cells were then seeded at 1 × 105 viable cells per well and the cultures were maintained in Neurobasal A medium (Life Technologies, Carlsbad, AC, USA) supplemented with 2% B27, 1% l-glutamine (2 mm: Life Technologies, Carlsbad, CA, USA) and 0.1% gentamicin (50 mg/mL: Life Technologies, Carlsbad, CA, USA).

Rat RGCs were seeded on confluent MG cultures from the center or periphery pig retina. In addition, RGCs were also cultivated adding either CM obtained from cultures of central or peripheral MG, collected in NBA plus NBA/B27 (1:1). As control, rat RGCs were cultured in NBA/B27. All the media used contained 1% l-glutamine and 0.1% gentamicin. RGC cultures were maintained for 6 days at 37 °C in a humidified atmosphere containing 5% CO_2_, and the medium was changed every 3 days. Finally, the RGCs were fixed for 10 min with methanol at − 20 °C on day 6.

At least four were performed for each analysis described, repeating each independent experiment a minimum of three times.

### Cultures exposed to high pressure

Cultures were subjected to high pressure (HP) using a custom-made humidified pressure chamber equipped with a pressure gauge and a pressure regulator as described previously with minor changes [30]. This set-up as allowed a constant pressure to be maintained with an air mixture of 95% air, 5% CO_2_ and 2%O_2_. The pressure chamber was placed in an oven at 37 °C and the cultures were maintained under HP for 72 h. The magnitude of pressure elevation (70 mmHg above atmospheric pressure) was chosen in accordance with previous studies [31]. Control cultures were kept at normal atmospheric pressure in a standard cell incubator, and at least three experimental replicates were analyzed, performed in triplicate independent experiments.

### Immunocytochemistry

After fixation in methanol and washing with PBS (phosphate buffered saline, pH 7.0), the binding of non-specific antigens was blocked with blocking buffer (3% BSA and 0.1% Triton X-100 in PBS) before incubating the cells overnight at 4 °C with the primary antibodies (see Table 1) diluted in blocking buffer. After washing, the binding of these antibodies were detected with Alexa Fluor 555 or 488 conjugated goat anti-mouse and goat anti-rabbit antibodies (diluted 1:1000: Life Technologies, Carlsbad, CA, USA). Cells were finally counterstained with the nuclear marker DAPI (diluted 1:10,000: Life Technologies, Carlsbad, CA, USA). After washing, the coverslips were mounted using Fluor-save Reagent (Sigma-Aldrich, St. Louis, MO, USA).

**Table 1:** Primary antibodies used

Antigen	Target	Host	Dilution	Supplier
α-SMA	Dedifferentiation	Mouse	1:1000	Abcam
β-III-Tubulin	RGCs	Rabbit	1:2000	Promega
β-Catenin	Dedifferentiation	Rabbit	1:2000	Abcam
CD-133	Dedifferentiation	Rabbit	1:200	Abcam
Nestin	Dedifferentiation	Rabbit	1:500	Sigma
OCT4	Dedifferentiation	Rabbit	1:200	Abcam
p75NTR	Müller glia	Rabbit	1:2000	Abcam
Piezo1	Mechanosensor	Rabbit	1:100	Life Technologies
Trpv4	Mechanosensor	Rabbit	1:500	Life Technologies
Vimentin	Müller glia	Mouse	1:10,000	Dako

*αSMA* α-smooth muscle actin, *TRPV4* transient receptor potential cation channel subfamily V member 4

### Quantification of RGCs and MG

RGCs were observed in an epifluorescence microscope (Zeiss, Jena, Germany) coupled to a digital camera (Zeiss Axiocam MRM, Zeiss, Jena, Germany). All images were obtained under the same conditions of intensity and exposure time. At least three coverslips were analyzed for each experimental condition and from a minimum of three independent experiments. The density of the RGC cultures was quantified. In addition, the RGCs were classified as: (1) cells with no neurites; (2) cells with a longest neurite < 50 μm; (3) cells with the longest neurite between 50 and 200 μm; and (4) cells with neurites longer than 200 μm. The total number of RGCs surviving in each condition was counted. The MG present in the cultures were also analyzed and counted in images. Semi-automatic Zen software (Zeiss, Jena, Germany) was used to count the number of nuclei stained with DAPI, taking into consideration the limits of the axis of the MG nuclei to obtain more accurate measurements. As such, we used a macro designed to specifically measure the limits of the axes (55–70 μm), which was corrected manually for each image.

### Mass spectrometry analysis of the CM and data processing

The proteomic analysis of the CM obtained of the central and peripheral MG cultures under control and HP conditions was carried out at the CIC bioGUNE Proteomics Service (Derio, Bizkaia, Spain), using the Filter Aided Sample Preparation (FASP) protocol [32], with minor modifications. After solution digestion, the proteins were extracted in a sample containing 7 M urea, 2 M Thiourea, 4% CHAPS and 5 mM DTT. Trypsin was added at a trypsin: protein ratio of 1:20, and the mixture was incubated overnight at 37 °C, dried in a RVC2 25 speedvac concentrator (Christ, Osterode am Harz, Germany), and resuspended in 0.1% formaldehyde (FA). Peptides were desalted and resuspended in 0.1% FA using C18 stage tips (Merck Millipore, Burlington, MA, USA). The samples were analyzed in a timsTOF Pro with PASEF (Bruker, Billerica, MA, USA) apparatus coupled online to a Evosep ONE liquid chromatograph (Evosep Biosystems, Odense, Denmark), loading 200 ng directly onto the Evosep ONE and employing a 60 samples-per-day protocol.

Protein identification and quantification was carried out using PEAKS X software (Bioinformatics solutions, Waterloo, Canada), carrying out searches against a database consisting of Sus scrofa entries from UniProt (https://www.uniprot.org/), with precursor and fragment tolerances of 20 ppm and 0.05 Da. Only proteins identified with at least two peptides at a False Detection Rate (FDR) of 1% were considered for further analysis. Protein abundances inferred from PEAKS were loaded onto the Perseus platform, log2 transformed and imputed before analyzing with a Student’s t-test.

Proteins that were considered significantly different between the groups were those with a p-value < 0.05, and also those that exceeded that value, up to a p-value of 0.1, but with a fold change > 2 for each comparison analyzed. The proteins listed were ordered according to the fold change obtained, and the proteins selected, out of a total of 893 proteins, were categorized based on their functions attributed in the UniProt database. In addition, for some proteins of particular interest in this study, an in silico analysis was carried out using the freely available STRING (Search Tool for the Retrieval of Interacting Genes/Proteins) database (https://string-db.org/). The number of protein–protein interactions registered in the database was determined for the proteins that were differentially overexpressed. For visualization, a diagram was assembled linking the proteins depicted by nodes based on recognized connections with the proteins identified.

### Statistical analysis

The experimental procedures were replicated at least three times to ensure the reliability and consistency of the findings. The cell density was defined as the mean number of cells per cm^2^, and the mean and standard error of mean (SEM) are presented for each condition. Statistical analyses were carried out using the IBM SPSS Statistical software v.24-0. The data from the different experimental conditions were compared using the non-parametric Mann–Whitney U test. When more than two independent groups were compared, a Kruskal–Wallis non-parametric test was used, and if the Kruskal–Wallis test was significant, a post-hoc Dunn test was performed in order to determine which groups differ from the others. Differences were considered significant for all tests at a p-value < 0.05.

## Results

**Fig. 1:**
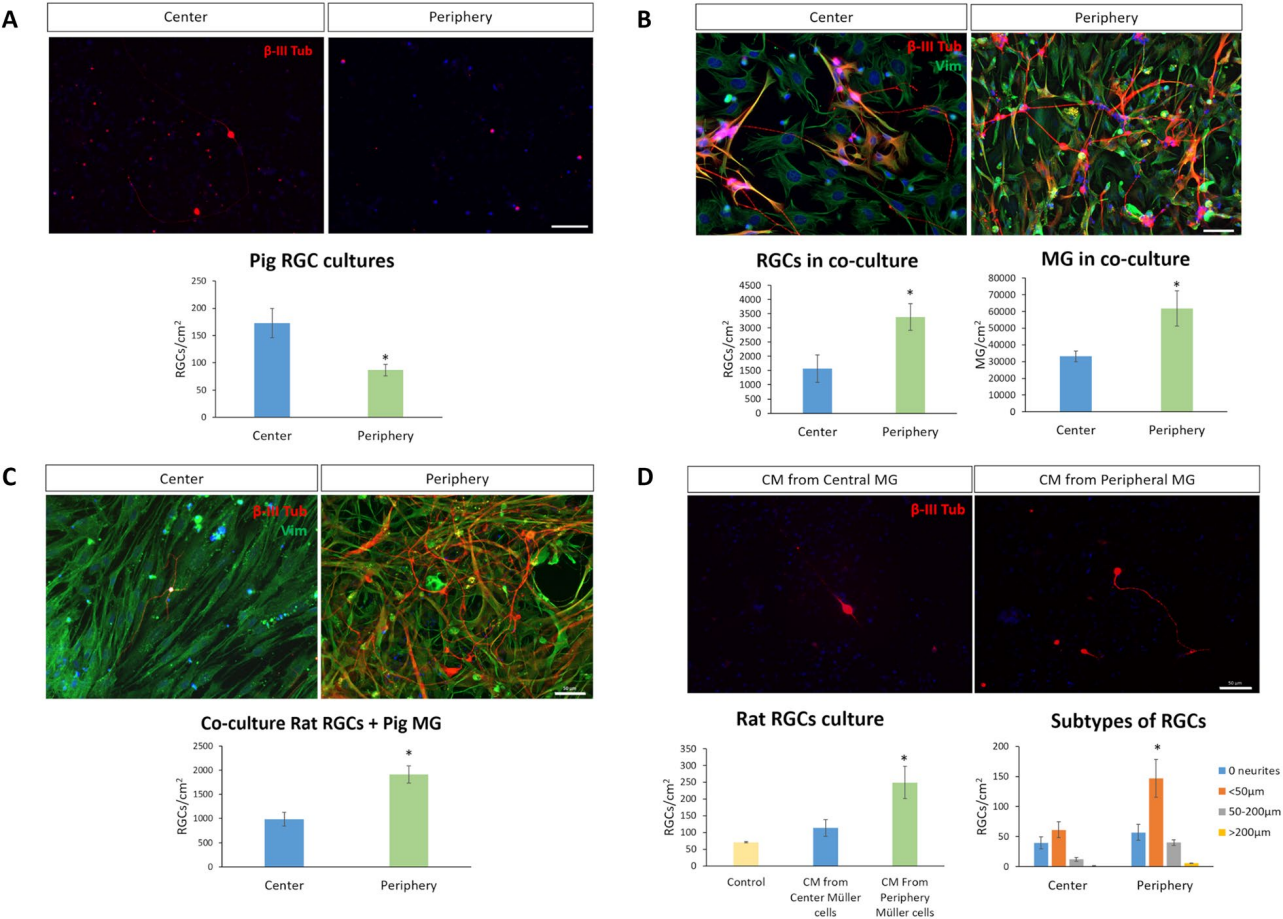
**A** The survival of central or peripheral pig RGCs in purified cultures. Images of purified RGC cultures from the central and the peripheral retina. The same number of RGCs were seeded in both cases. Histogram of the number of RGCs in the cultures from each retinal region. **B** Effect of MG from the central and peripheral retina on the survival of RGCs from the central and peripheral retina, respectively, in co-cultures. Images of co-cultures of pig RGCs and MG from the central and peripheral retina. The MG and RGC in the co-cultures were both isolated from the same area of the retina (center and periphery). Number of RGCs in co-cultures from the center and periphery of the retina and number of MG in co-cultures from the center and periphery of the retina were represented. **C** The survival of rat RGCs seeded on confluent pig MG isolated from the central and peripheral retina. Images of rat RGCs seeded on pig MG from the central and peripheral retina. The same initial number of rat RGC were seeded on pig MG cultures. The number of rat RGCs present on pig MG cultures from the central or peripheral retina. **D** Survival and neuritogenesis of rat RGCs when maintained in CM secreted by MG from the central and peripheral retina. Images of rat RGCs maintained with conditioned medium (CM) from central and peripheral pig MG. The same number of RGCs were seeded in all conditions. Number of surviving rat RGCs maintained with CM secreted by MG from the central or peripheral pig retina was represented. To analyze neuritogenesis, the RGCs were classified as RGCs without neurites (blue), RGCs with the longest neurite < 50 μm (orange), RGCs with the longest neurite between 50 and 200 μm (grey), and those with neurites longer than 200 μm (yellow). The number of RGCs in each category is shown for those maintained in the presence of both types of CM. The RGCs were labelled with antibodies against β-III-Tubulin (red) and the MG with vimentin (green). Nuclei were stained with DAPI (blue): *p-value < 0.05. Scale bar: 50 µm

The survival of pig RGCs from the center or from the periphery of the retina was analyzed in purified cultures. Cultured RGCs from the central retina survived significantly better (173 ± 27 RGCs/cm^2^) than those from the peripheral retina (87 ± 11 RGCs/cm^2^, Fig. 1A). When the effect of MG, isolated from the center or periphery of the pig retina, on RGC survival was analyzed in co-cultures, the survival of peripheral RGCs and MG (3389 ± 471 and 61,876 ± 10,509 cells/cm^2^, respectively) was significantly increased comparing to co-cultures with MG and RGCs isolated from the central retina (1566 ± 484 RGCs/cm^2^ and 33,232 ± 3181 MG/cm^2^: Fig. 1B). To confirm the more robust neuroprotective effect of the MG isolated from the peripheral retina, purified rat RGCs were seeded onto a monolayer of MG from the central and peripheral retina already cultured for 7 days in vitro (DIV). The survival of the rat RGCs increased significantly (1914 ± 176 RGCs/cm^2^) when they were seeded on peripheral MG rather than on central retinal MG (987 ± 139 RGCs/cm^2^) after 6 days in co-culture (Fig. 1C). To assess whether factors secreted by peripheral MG may have the same effect on RGC survival as MG monolayers, rat RGCs were cultured in CM from the central and peripheral MG. RGCs were cultured in NBA/B27 (control) or in NBA/B27 medium:CM (1:1) obtained from either central or peripheral MG in culture. More RGCs survived when the cells were maintained in CM from peripheral MG (249 ± 48 RGCs/cm^2^) than in the CM from central retinal MG (114 ± 25 RGCs/cm^2^), or control RGCs without CM (71 ± 2 RGCs/cm^2^) (Fig. 1D).

The RGC neurite length was assessed to determine whether the different CM affected these parameters (Fig. 1D). When RGCs were maintained in CM secreted by peripheral MG, there were more RGCs with short neurites (< 50 μm, 147 ± 32 RGCs/cm^2^) than when RGCs were maintained in CM by MG from the central retina (61 ± 13 RGCs with short neurites/cm^2^).

**Fig. 2:**
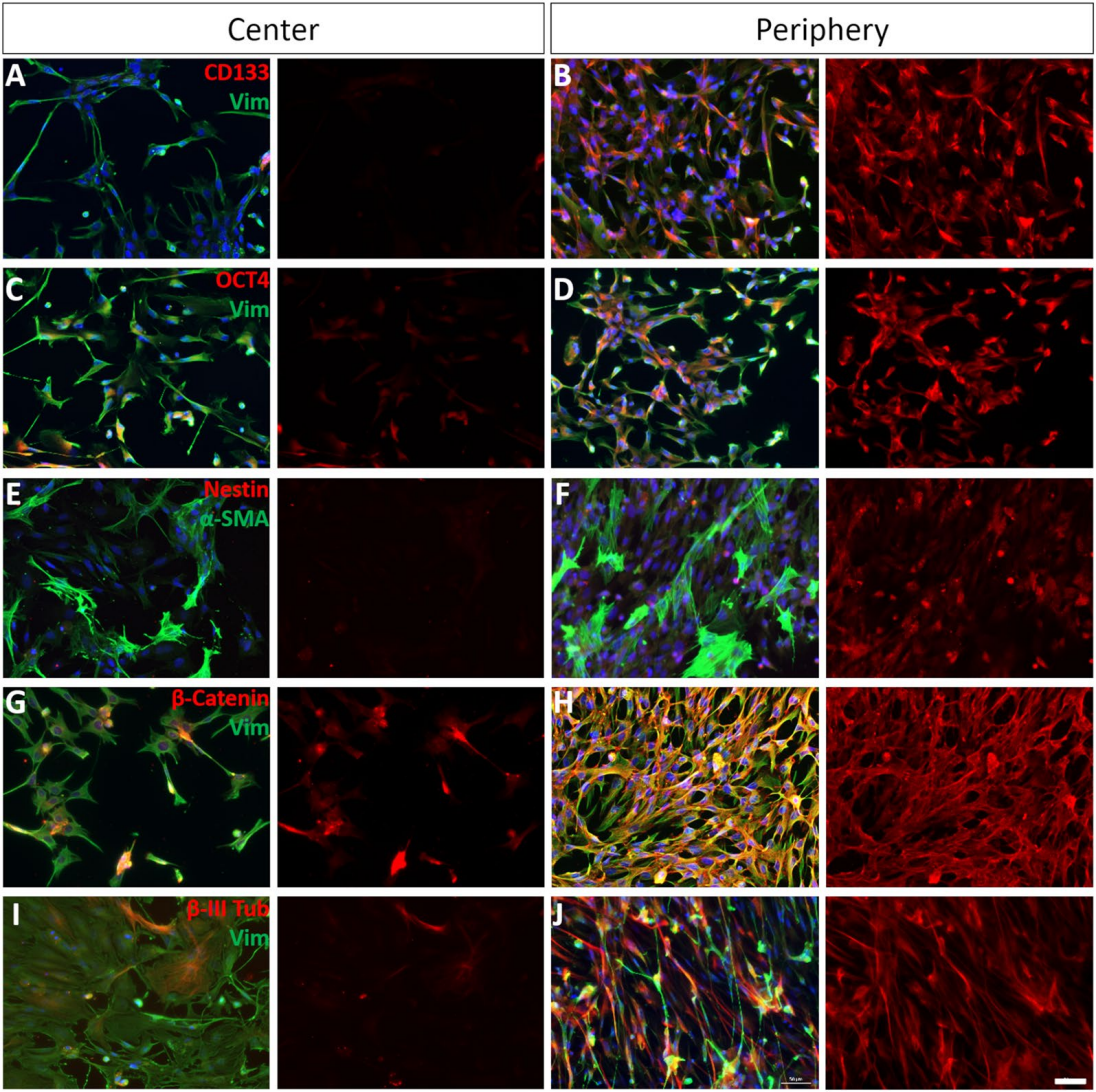
Immunolabelling of vimentin, CD133, OCT4, nestin, α-SMA, β-catenin and β-III Tubulin in pure cultures of MG isolated from the central and peripheral pig retina. Images of cultured MG isolated from the center (**A**, **C**, **E**, **G**, **I**) or periphery (**B**, **D**, **F**, **H**, **J**) of the retina. The cells were labelled with antibodies against CD133 (**A**, **B**, red), OCT4 (**C**, **D**, red), nestin (**E**, **F**, red), β-catenin (**G**, **H**, red), β-III Tubulin (**I**, **J**, red), α-SMA (**E**, **F**, green), or vimentin (**A**–**J**, green). Nuclei were stained with DAPI (blue). Note the increase in the expression of all stem cell-like markers and β-III-Tubulin in the peripheral MG. Scale bar: 50 µm

To determine if the central or peripheral MG behave differently in culture, the expression of certain stem cell-like markers was analyzed in purified MG cultures: vimentin (MG specific marker), β-III-Tubulin (neural marker), α-SMA (dedifferentiation marker), CD133 (glial stem cell marker), OCT4 (important in MG reprogramming during retinal regeneration in zebrafish), nestin (marker for glial and neuronal progenitors), and β-catenin (stem cell-like marker). Although the MG specific marker vimentin was expressed strongly in both types of cultures, β-III-Tubulin, α-SMA, CD133, OCT4, nestin, and β-catenin were expressed more by MG from the peripheral retina than by central MG. Hence, peripheral MG appear to be more dedifferentiated towards a stem cell-like phenotype (Fig. 2).

**Fig. 3:**
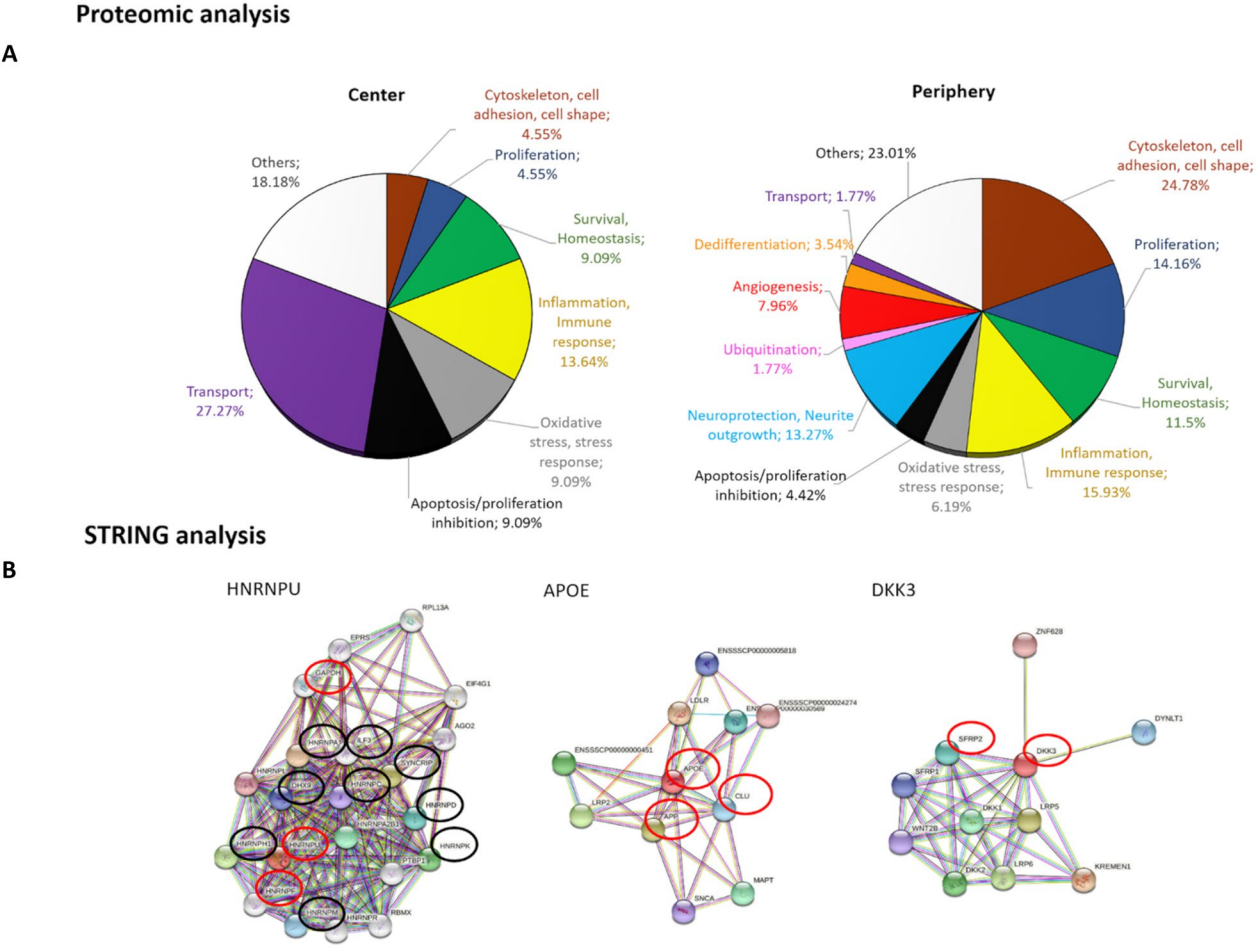
Functional and STRING analysis of the proteins in the MG CMs. **A** Graphical representation of the principal functions of the proteins most strongly represented in the CM from central and peripheral MG cultures. **B** STRING analysis of three proteins reflecting the principal activities of the proteins in the peripheral MG CM: HNRNPU regulates cell proliferation, APOE is known for its neuroprotection, and DKK3 is a Wnt pathway regulator promoting dedifferentiation in MG. The red and black circles represent other proteins identified in the proteomic analysis of the CM. The red circles are proteins more significantly expressed in the CM from peripheral MG and the black circles are proteins in peripheral CM with at least a twofold increase relative to the CM from the central MG

The analysis of the proteome of CM obtained from the central and peripheral MG was performed, identifying the expression of more stem cell-like markers in the CM of peripheral MG, and also more neuroprotective molecules. The proteome of the MG CMs contained differentially expressed proteins (DEPs), with 22 more DEPs in the central MG CM and 113 proteins more strongly expressed in the peripheral CM. All proteins more strongly expressed in the CM of peripheral MG, along with their associated functions: “Cytoskeleton, cell adhesion and cell shape”; “Inflammation and immune response”; “Survival and homeostasis”; “Neuroprotection and neurite outgrowth”; “Proliferation”; “Ubiquitination”; “Angiogenesis”; “Dedifferentiation”; “Transport”; “Apoptosis and proliferation inhibition”; “Oxidative stress and stress response”; and “Other functions” (Table 2). When the proteins sorted by function were compared between central and peripheral CM (Fig. 3A), the proportion of proteins in the peripheral CM related to “Proliferation” and “Cytoskeleton, cell adhesion, cell shape” were 14.2% and 24.8% of total proteins identified, respectively, while in central CM only 4.6% of proteins were related to these functions. Another interesting function for the present study is “Neuroprotection and neurite outgrowth” and as expected, the proteins related to this role were more strongly expressed in the peripheral CM, representing 13.3% of the proteins. Another function worth highlighting is “Dedifferentiation” and although the proportion of these proteins was not very high (3.5%), they only appeared in the peripheral CM, consistent with the expression of the stem cell-like markers detected (Fig. 2).

**Table 2:**
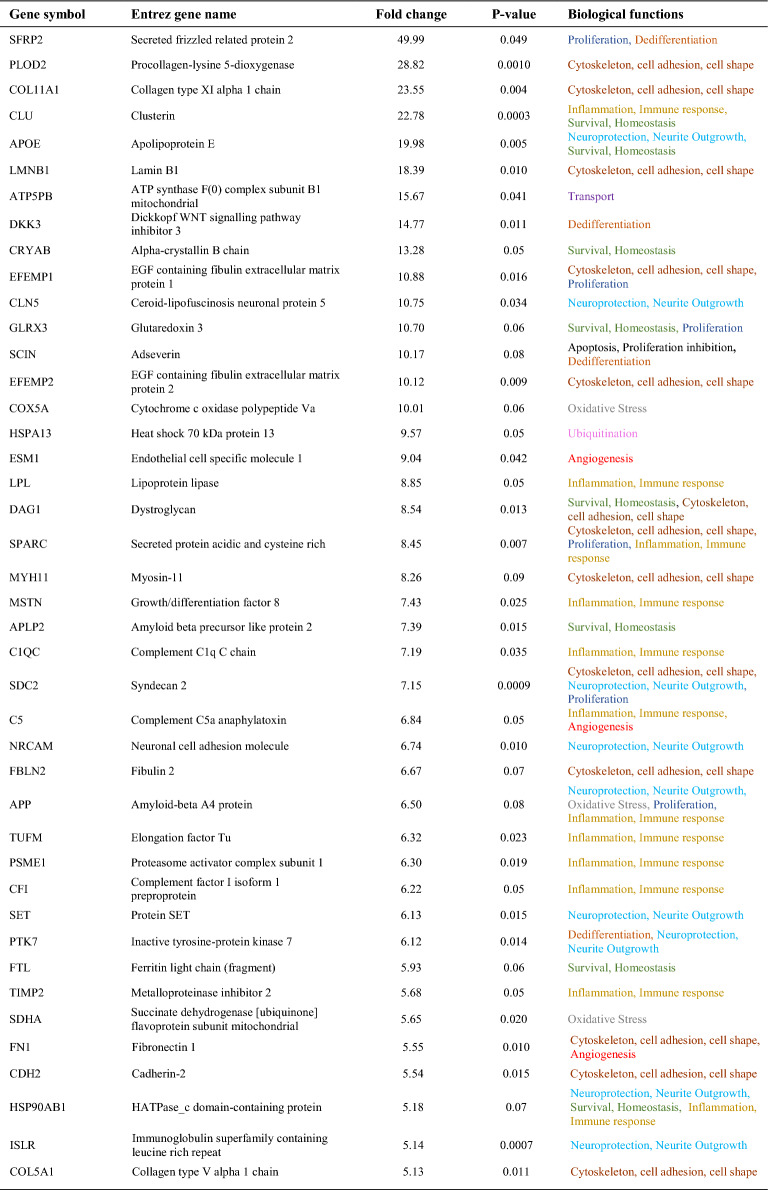

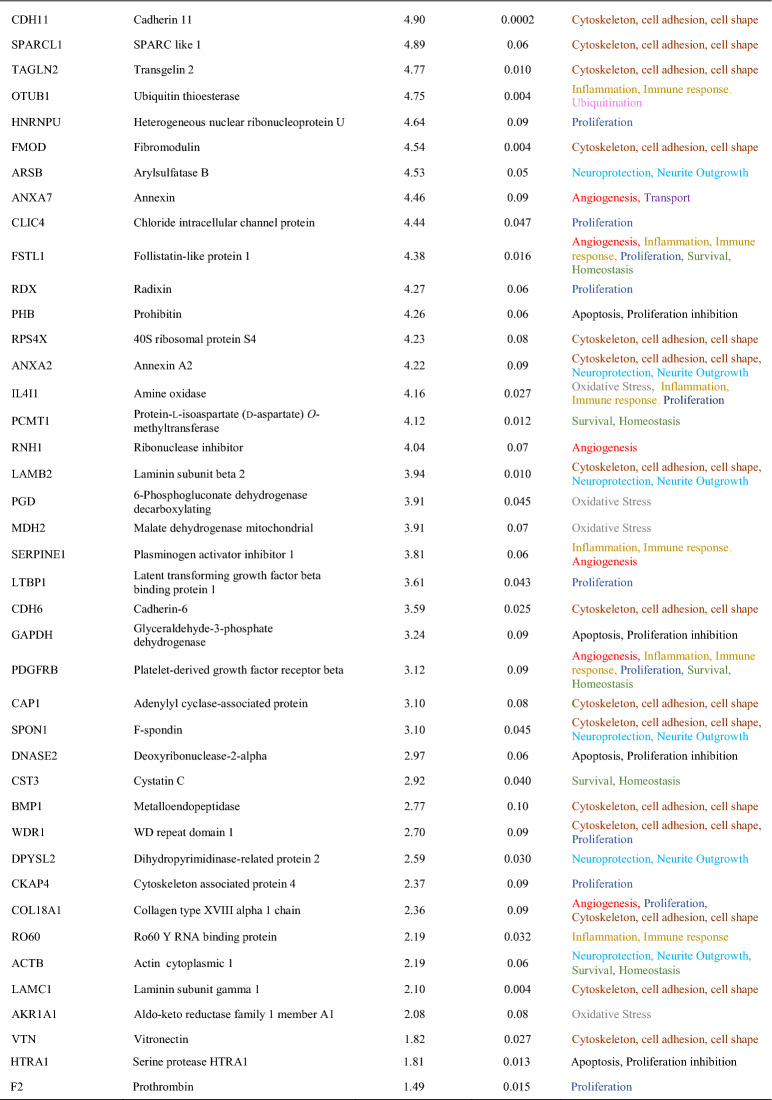
Proteins more strongly represented in the CM from peripheral MG comparing to the central MG, and their functions

The proteins mentioned in the table are a selection of more than 890 proteins obtained in the proteomic analysis. Proteins considered significantly different between groups were those with a p-value less than 0.05 and that exceeded that value but had a > twofold change in each comparison analyzed, up to a p-value of 0.1. Proteins listed were ordered by the fold change observed

A STRING analysis was also performed on some of the proteins from the “Proliferation”, “Neuroprotection and neurite outgrowth” and “Dedifferentiation” categories identified in our proteomic analysis. Again, some of the proteins identified in the STRING analysis were more strongly represented in the peripheral CM (Fig. 3B).

**Fig. 4:**
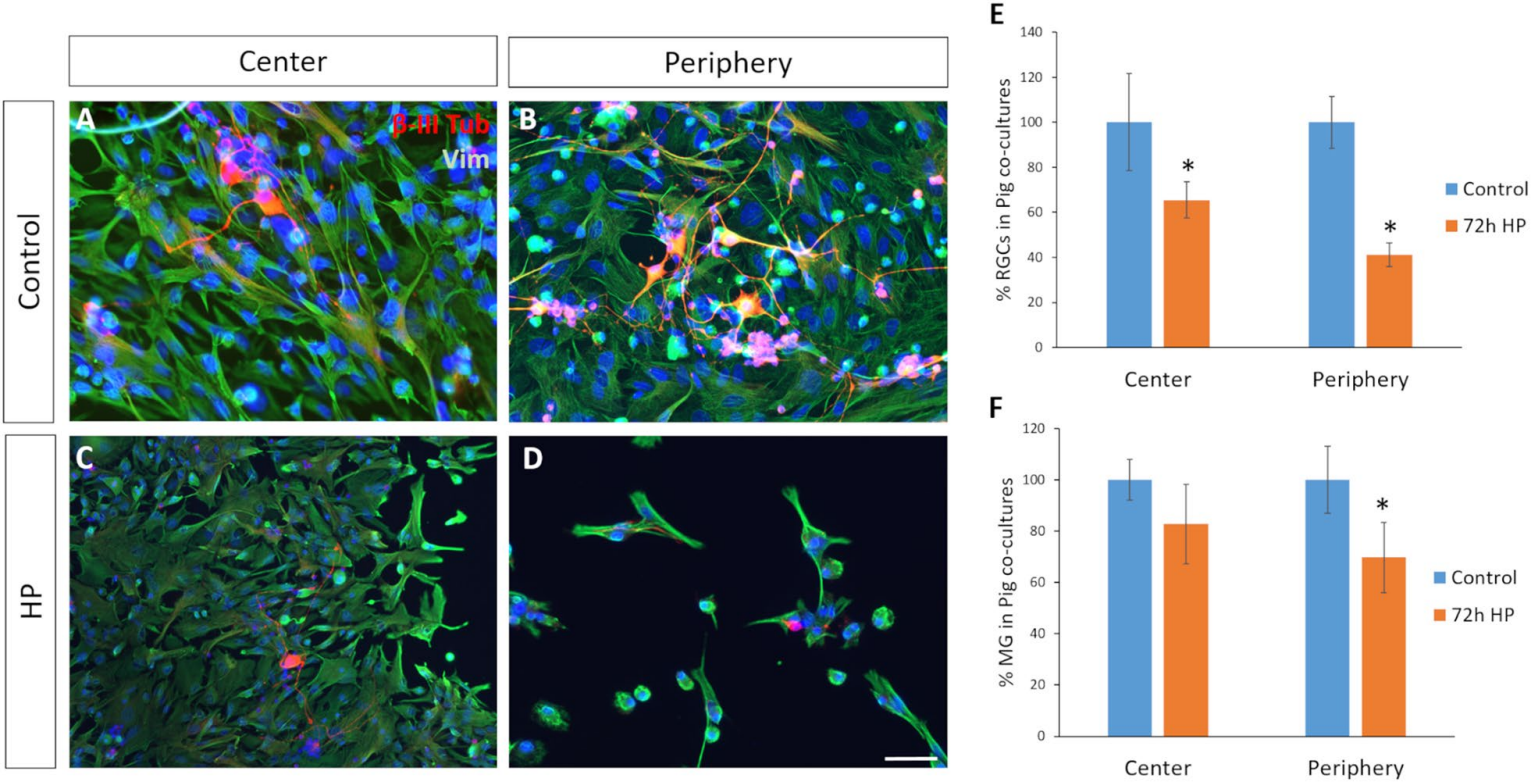
Effect of HP on the survival of central and peripheral pig RGCs and MG in co-cultures. Images of co-cultured pig MG and RGCs from **A** the center and **B** the periphery of the retina under control condition, and from **C** the center and **D** the periphery of the retina after 72 h of HP. The cells were labelled with antibodies against β-III-Tubulin (red) and vimentin (green). Nuclei are stained with DAPI (blue). **E**, **F** Number of central and peripheral RGCs (**E**) or MG (**F**) in the co-cultures under control conditions and HP: *p-value < 0.05; Scale bar: 50 µm

The effect of HP on RGC and MG survival was analyzed in co-cultures from the central and peripheral retina. After 72 h of HP, central RGC survival decreased to 65.5 ± 8.0%. Surprisingly, the survival of peripheral RGCs was more strongly affected, decreasing to 41.2 ± 5.3% relative to the control. The same applied to the MG, with 82.7 ± 15. % of the central MG surviving after 72 h of HP, and decreasing to 69.6 ± 13.6% when the MG were from the periphery of the retina (Fig. 4).

**Fig. 5:**
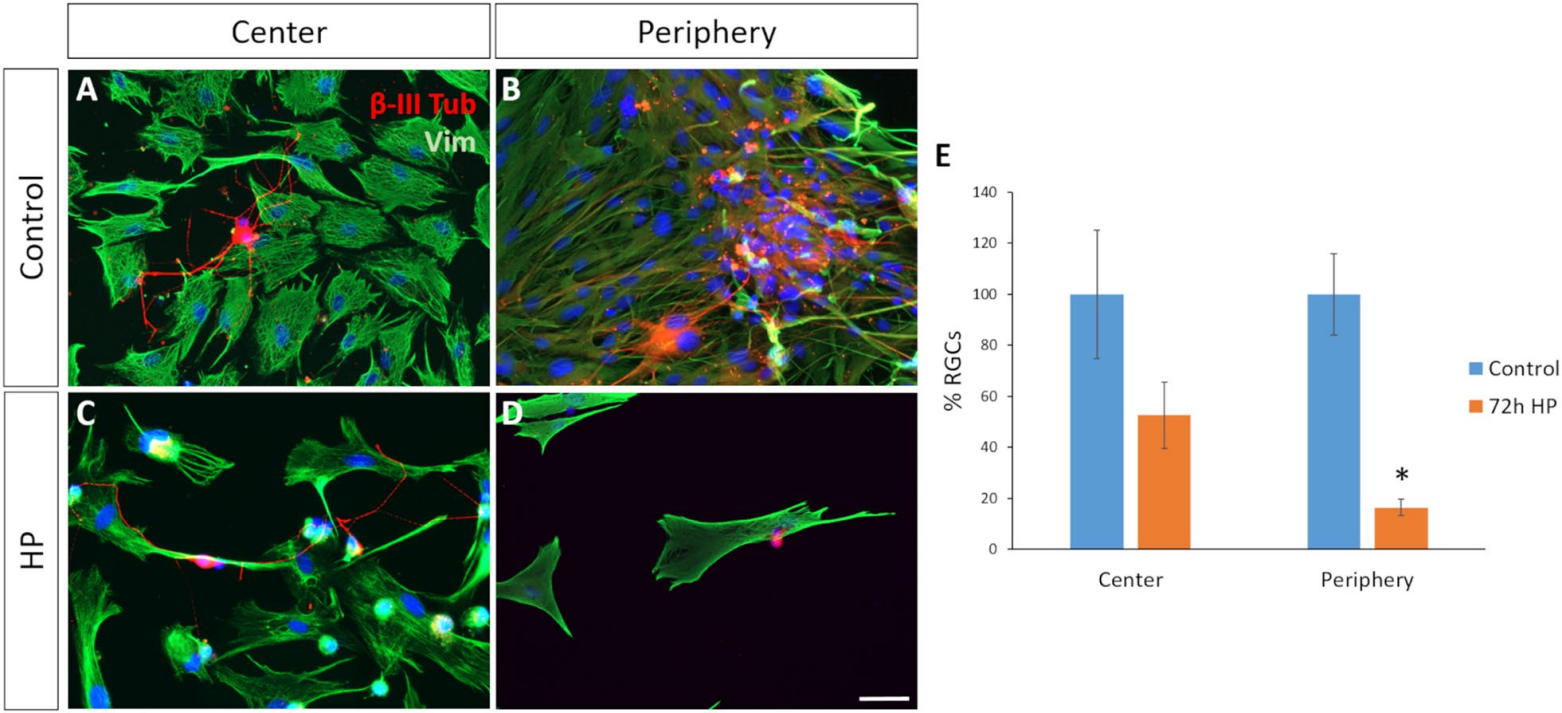
Effect of HP on the survival of rat RGCs seeded on pig MG from the central and peripheral retina. Images of co-cultured rat RGCs with pig MG from **A** the central and **B** the peripheral retina in control condition, and from **C** the central and **D** peripheral retina after 72 h exposure to HP. The cells were labelled with antibodies against β-III-Tubulin (red) and vimentin (green). Nuclei are stained with DAPI (blue). **E** Number of rat RGCs in co-cultures with pig MG from the central and peripheral retina in control and HP conditions: *p-value < 0.05; Scale bar: 50 µm

To confirm the effect of HP on the susceptibility of peripheral MG, purified control rat RGCs were seeded on a MG monolayer from the central or peripheral pig retina at 7 DIV, and exposed to HP for 72 h. The survival of the rat RGCs, comparing to the control, decreased dramatically when they were seeded on peripheral MG under conditions of HP (16.4 ± 4.5% RGCs) as opposed to MG from the central retina (52.6 ± 30.1% RGCs) (Fig. 5).

**Fig. 6:**
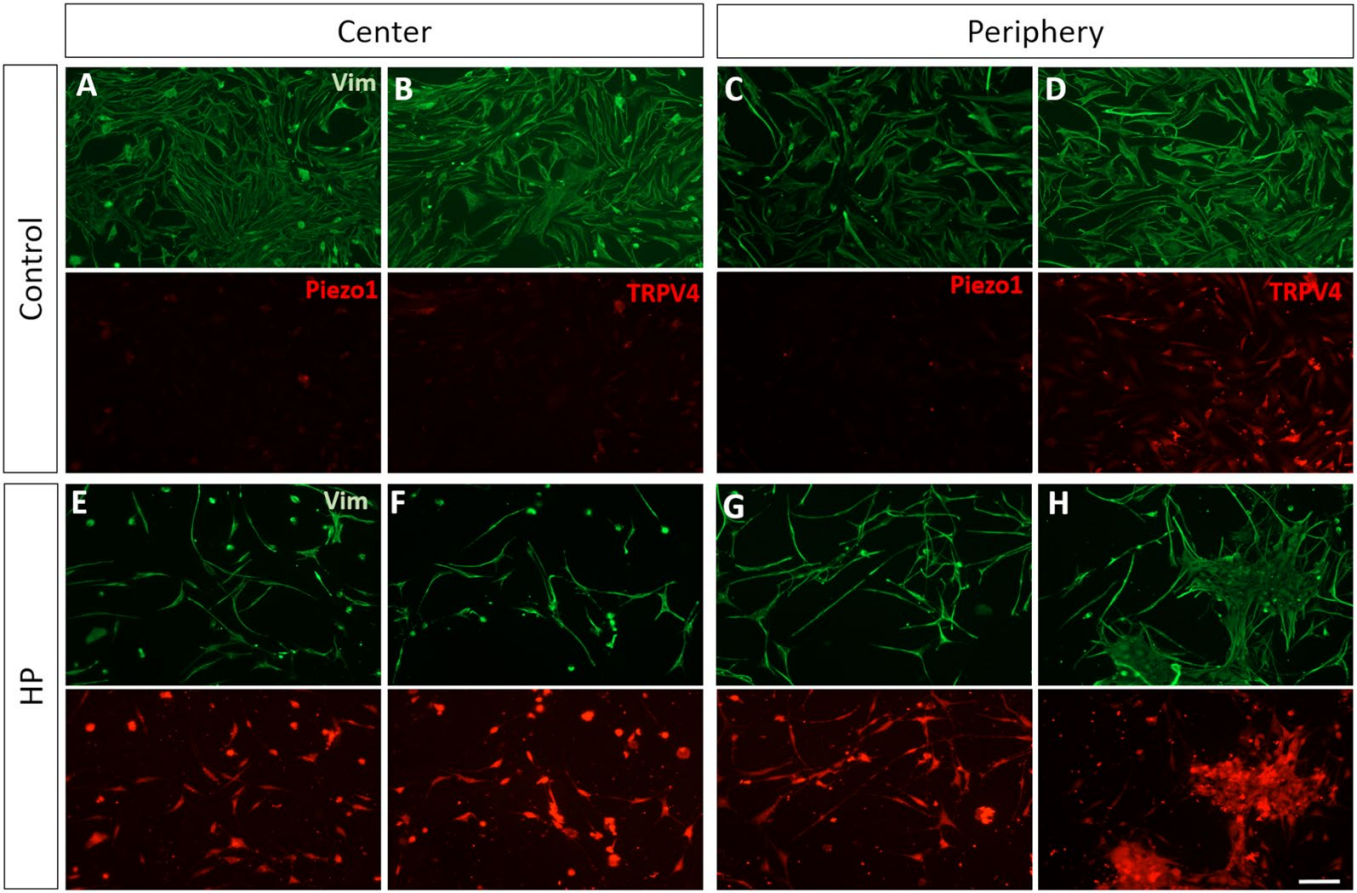
Immunolabelling of the pressure receptors PIEZO1 and TRPV4 in purified cultures of MG isolated from the central or peripheral pig retina under control conditions or HP. Images of cultured MG isolated from the central (**A**, **B**, **E**, **F**) and peripheral (**C**, **D**, **G**, **H**) retina in control conditions (**A**, **E**, **C**, **G**) and in response to EH (**B**, **F**, **D**, **H**). The cells were labelled with antibodies against PIEZO1 (**A**–**D**, red), TRPV4 (**E**–**H**, red) and vimentin (green). Nuclei were stained with DAPI (blue). Note the enhanced immunoreactivity of both pressure receptors in response to HP. TRPV4 is also expressed in the control peripheral MG. Scale bar: 50 µm

Having observed distinct susceptibility of both RGCs and MG to HP depending on their position in the retina, central or peripheral, we analyzed the expression of two common pressure receptors, PIEZO1 and TRPV4 in purified MG cultures under control conditions and after exposure to HP for 72 h. At 7 DIV, both central and peripheral MG express PIEZO1 in response to HP (Fig. 6E, G). Likewise, TRPV4 is also more expressed in central and peripheral MG when they are exposed to HP. In addition, in control condition, is more strongly expressed in peripheral MG than in the central MG (Fig. 6F, H).

**Fig. 7:**
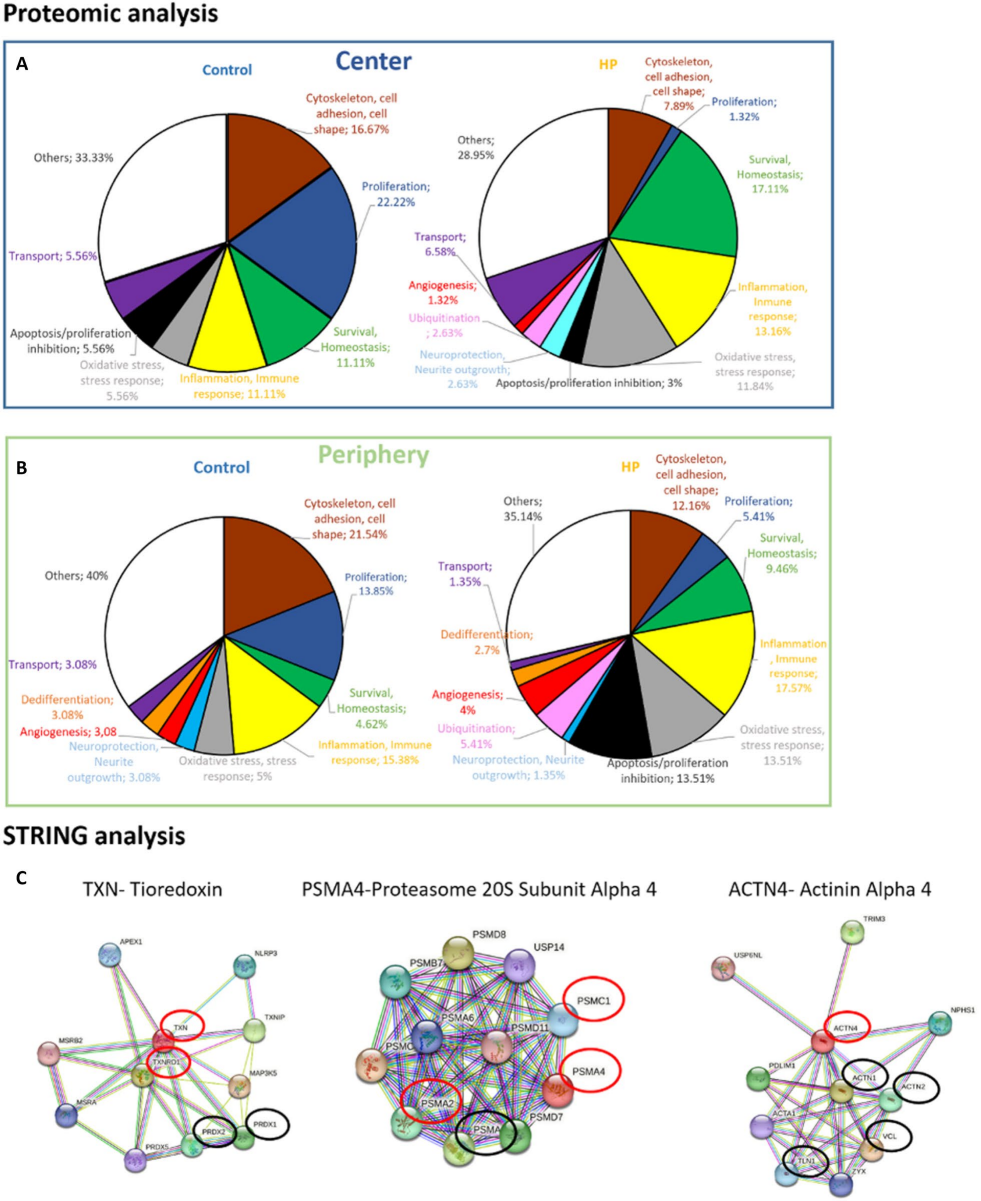
The functional analysis of the proteins in the CM derived from central and peripheral MG under control conditions and when exposed to HP, and a STRING analysis for some selected proteins. Graphical representation of the principal functions of the proteins more represented in the CM from central (**A**) and peripheral (**B**) pig MG cultures in control conditions and when exposed to HP. **C** STRING analysis of three proteins that were more strongly expressed in the peripheral CM under HP conditions. When considering all proteins obtained in the STRING analysis, circles surround the proteins identified in the proteomic analysis, with red circles around proteins that were significantly more expressed in the peripheral CM, and black circles represent those present in peripheral CM with at least a twofold increase relative to the control peripheral MG CM. The three proteins represent some of the principal functions of the proteins found in the peripheral HP CM: TXN mediates the response to reactive oxygen species, ACTN4 induces apoptosis, and PSMA4 is involved in inflammation-related pathways and networks

The proteome of the CM obtained from the central and peripheral pig MG exposed to HP and under control condition was compared. When the CM from central MG exposed to HP was compared to control, 18 proteins were more significantly expressed in the control CM while 75 proteins were significantly more expressed in the EH CM. When the proteins were sorted by function, there were more proteins related to “Oxidative stress, stress response” (11.8%) and “Survival, Homeostasis” (17.1%) in the EH CM comparing to the control CM, in which the percentage of proteins related to “Oxidative stress, stress response” and “Survival, Homeostasis” was 5.6% and 11.1%, respectively (Fig. 7A).

Having detected a greater vulnerability of both peripheral MGs and RGCs to HP, we focused closer on the protein content of the CM obtained from the cultured peripheral MG. When the CM from peripheral MG subjected to HP was compared to that obtained in control conditions, we detected 74 proteins more strongly expressed in the HP CM while 65 proteins were more strongly expressed in the control CM (Table 3). Of these proteins, “Dedifferentiation” proteins like DKK3 or nestin appeared in the peripheral CMs that were not evident in the CM from central MG under control or HP conditions. It was noteworthy to that there was an increase in proteins related to both “Oxidative stress, stress response” (13.5%) and “Apoptosis and proliferation inhibition” (13.5%) in the peripheral HP CM comparing to the control (5% and 0% of the proteins, respectively). Proteins related to “Inflammation, Immune response” were also more strongly represented in the HP CM (17.6%) than in control CM (15.4%: Fig. 7B). Hence, these data could explain the responses of RGCs and MG to HP (Figs. 5 and 6), which enhanced the death of both cell types in peripheral cultures. The complete proteomic analysis and comparisons is available in Additional file 1.

**Table 3:**
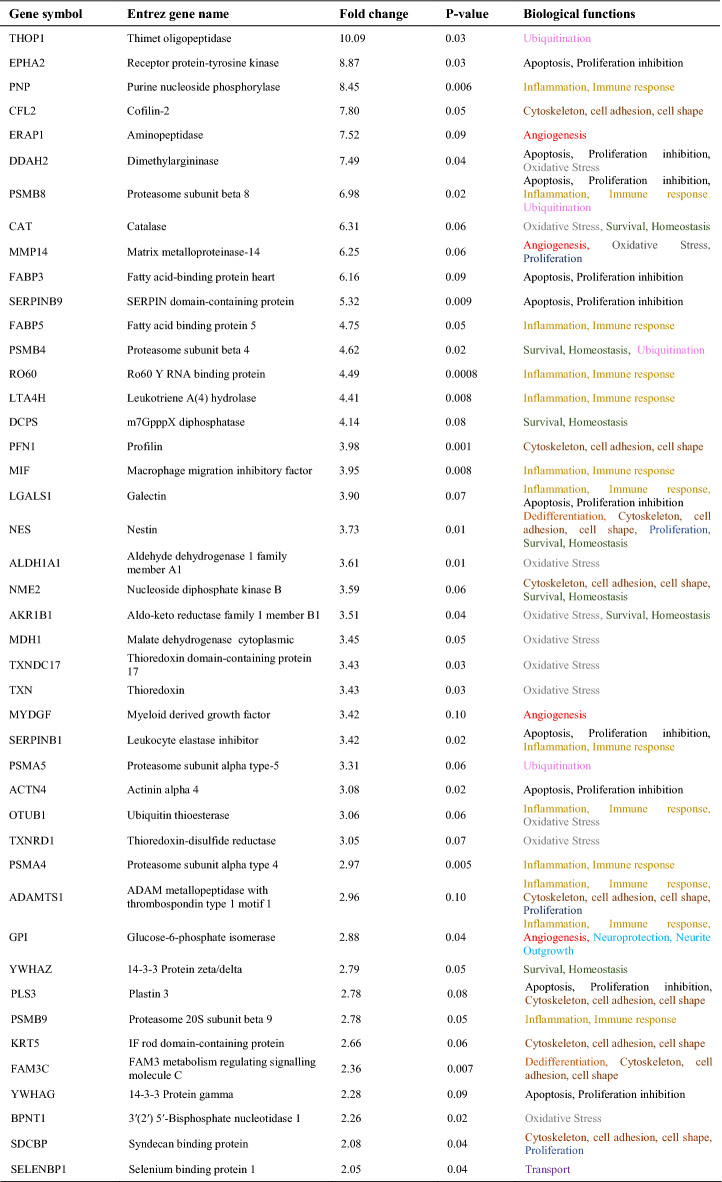
Changes in the expression of key proteins in the CM from peripheral MG exposed to HP compared to control peripheral MG CM, and their functions

The proteins represented in the table are a selection of more than 890 proteins obtained in the proteomic analysis. Proteins that were considered significantly different between groups were those with a p-value of < 0.05, or proteins that exceeded that value but had at least a twofold increase in each comparison and up to a p-value of 0.1. The proteins listed were ordered in the order of the fold change observed

A STRING analysis was also performed in some of the proteins from the principal functions found in the analysis of the proteome obtained: “Oxidative stress, stress response”, “Apoptosis and proliferation inhibition” and “Inflammation, Immune response”. The proteins that resulted from these analyses were searched in our database from proteomic analysis. The STRING analysis of three proteins reflecting each of these three functions were presented. We found that some of the proteins that interconnect in the STRING analysis appeared in our database, and these proteins were also more strongly represented in the peripheral CM exposed to HP (Fig. 7C).

## Discussion

Protecting neurons when their survival is compromised is a good strategy to tackle nervous system lesions and neurological disorders [33]. In glaucoma, considered a neurodegenerative disease, it has become crucial to better understand the fundamental role of glial cells, like MG, and their functional heterogeneity, which may open up novel therapeutic avenues [34]. Here, we have analyzed the neuroprotective effect of MG and their susceptibility to HP based on their location in the retina. We used the pig retina to study the susceptibility of RGCs to death as the pig and human eyes are very similar, being comparable in size and retinal topography [35]. Moreover, we previously demonstrated that pig RGCs die in models of glaucoma in a similar pattern to humans, starting in the peripheral retina [6]. Besides, neurons and glia cultured in vitro retain their neurotrophin and neurotrophin receptors expression in vivo [36], and MG preserve their neuroprotective capacity in vitro [11]. Previous studies of the group also analyze the proteomics of pig MG [10], and their responses to different insults and culture conditions [12, 37, 38], as well as in culturing RGCs and MG from other animals [28]. These in vitro models have become a great strategy to study the role of MG in RGC survival and the behavior of both cell types in glaucoma models. Here we report that the neuroprotective effect of MG is a general characteristic of these cells, which varies depending on their location within the retina, with MG located peripherally exerting a stronger neuroprotective effect than those located centrally.

Since there are more MG in the center of the retina, we expected a higher number of MG in the cultures from this region. However, culturing the same surface area of the retina, after 7 DIV there were more MG in the peripheral cultures than in cultures of central MG, since in the peripheral retina, more MG cells divide. This different capacity of central and peripheral MG to divide is consistent with the proteomic analysis presented here, since there are more proliferation-related proteins in the CM of peripheral MG than in central MG.

The enhanced neuroprotective effect of peripheral pig MG was not only effective in co-cultures with pig RGCs, but also when they were co-cultured with rat RGCs, confirming that pig MG secrete common neuroprotective factors despite being in contact with RGCs from other species, as seen previously [10]. Indeed, while MG express neuroprotective factors on their surface that act through contact, they also exert this effect by secreting factors into their *milieu* [10–12]. Thus, when the effect of CM produced by MG on cultured rat RGCs was assessed, a higher neuroprotection of RGC was evident when culturing RGCs with CM from peripheral MG, again indicating that the neuroprotective capacity of peripheral MG is stronger than that of MG from the central retina.

Markers of dedifferentiation or stem cell-like properties were more expressed in MG isolated from the peripheral retina, consistent with the acceptance that the periphery of the retina is less differentiated [17], as particularly evident in fish [39] and to some extent in humans [40]. Indeed, peripheral MG can express markers of dedifferentiation in vivo [21, 22]. MG in vitro can rapidly change their protein expression and adopt a fibroblast-like phenotype [41]. To avoid this phenotypic dedifferentiation change our cultures never exceeded more than 15 DIV. As the neuroprotective capacity can be related to differences in their dedifferentiation capacity, we analyzed the expression of specific markers of MG, like vimentin [16], neurons (β-III-Tubulin) [42], stem cell-like or progenitors (CD133, OCT4, β-catenin and nestin), and fibroblasts (α-SMA) [43]. On the same DIV, MG from each retinal region expressed vimentin, but notably the rest of the aforementioned markers which are directly associated with cellular pluripotency [44], were clearly more expressed in the peripheral MG, suggesting they are in a less differentiated state. It is worth mentioning that dedifferentiation or pluripotentiality may be directly related to increased cell proliferation. Proliferation, akin to pluripotency, constitutes a significant aspect of cellular stemness [45].

Interestingly, previous studies with dedifferentiated cells (e.g. mesenchymal stem cells) showed that they can protect RGCs by releasing neurotrophic and neuroprotective factors [46–48]. Accordingly, the more dedifferentiated the state of peripheral MG, might provoke the release of more neurotrophic and neuroprotective factors, enhancing RGC survival. We observed notably stronger β-catenin expression in peripheral MG, which could explain their enhanced neuroprotection relative to central MG. β-catenin is an integral structural component of cadherin-based adherens junctions and the key nuclear effector of canonical Wnt signaling in the nucleus [49]. Proteins associated with canonical Wnt signaling are expressed in the neuroretina and these signals are active in RGCs, MG, microglia and amacrine cells [50]. The activation of Wnt signaling in MG enhances photoreceptor survival and function [51], RGC survival [52] and neurite outgrowth [53], indicating an important neuroprotective effect of Wnt signaling in MG.

The proteomic analysis of MG CM helped to identify the factors secreted by these cells, which can help to understand the results here obtained. Comparing the CMs obtained from central and peripheral MGs, certain proteins were more expressed in one or other cell type. When sorted by function, the main functions among the most strongly expressed proteins in the peripheral CM were “Proliferation”, “Neuroprotection and Neurite Outgrowth” and “Dedifferentiation”, consistent with the results obtained. For example, some proteins were related to enhanced proliferation, such as HNRNPU that is a key regulator of the cell cycle [54]. We also found proteins related to neuroprotection, like: APOE, the major apolipoprotein in the CNS that is associated with anti-inflammatory and anti-apoptotic effects [55]; SPON1, also known as F-spondin, a secreted extracellular matrix glycoprotein that promotes neural cell adhesion, neuronal survival and outgrowth [56]; and CRYAB, that offers neuroprotection to RGCs [57]. It is also important to mention the protein clusterin, known for its neuroprotective effect, which is much more expressed in the peripheral MG, and that was previously shown to be secreted by primary pig MG and to have a neuroprotective effect on RGCs [10].

It is also important to note that only in the peripheral CM appear stem cell-like or dedifferentiation proteins among the proteins mainly expressed, which is in accordance with the immunocytochemical data. For example, Dkk3 is a member of the Dickkopf (Dkk) family of proteins, a cell-specific positive regulator of the canonical Wnt-β-catenin signaling. In the retina, Dkk3 is strongly expressed by MG and RGCs during retinal development [58]. These findings are consistent with increased β-catenin expression by peripheral MGs in vitro. The STRING analysis highlights protein–protein interaction networks, which is important for the system-level understanding of cellular processes. This analysis is based on physical interactions and on the interactions of different molecular pathways. The STRING analyses represented here focused on proteins that are more strongly represented in the CM of the peripheral MG, highlighting interactions with proteins mainly represented in this CM and supporting the results obtained in the proteomic analysis. These signaling pathways may underlie the differences between the two types of MG and their effect on RGC survival.

Furthermore, elevated IOP is the main risk factor for the onset and progression of glaucoma. Although there has been considerable research in the field of glaucoma, the pathological mechanisms underlying the disease onset and development are still not fully understood. Neuronal degeneration in glaucoma might be due to a combination of factors, among which the RGC and MG interactions. The effect of HP mimics the effect of elevated IOP in the retina [59]. In primary cultures of MG and RGCs exposed to HP, the cell death is enhanced [60]. Moreover, retinal astrocytes and microglia have a differential effect on the pressure-induced death of RGCs [61]. The obtained results demonstrate that peripheral and central MG have different susceptibility to HP, and this impacts on the type of secreted factors, consequently affecting RGC survival.

It is known that MG are sensors of pressure within the retina, as demonstrated in vivo [27] and in vitro [62]. We studied how conditions of HP might alter the expression of the pressure receptor channels TRPV4 and Piezo1, demonstrating that both receptors were overexpressed in MG exposed to HP. Piezo proteins play important roles in touch sensing pressure, respiration, angiogenesis and stem cell differentiation, and their activation increases calcium influx raising the intracellular calcium ion concentration ([Ca^2+^]i), which may trigger apoptosis [63]. Therefore, changes in cell stiffness and pressure acting through Piezo1 mechanosensitive channels could contribute to neurodegeneration [64]. TRPV4 activation can also provoke an increase in [Ca^2+^]i and continued channel activation induces MG gliosis in the mouse retina, as well as apoptosis in cultured mouse RGCs and adult porcine RGCs [65–67], which is in accordance with the data presented here. Indeed, the TRPV4 expression in peripheral MG could explain why these glial cells are more susceptible to HP.

The proteomic analyses of the central and peripheral CM from control and HP conditions identified a series of DEPs. When these were sorted by function, an increase of proteins related to “Oxidative stress and stress response”, “Inflammation and immune response” and “Apoptosis and inhibition of proliferation” was evident in the CM from the peripheral MG exposed to HP. However, in the CM of central MG there was an increase of the proteins related to “Survival and homeostasis”. These results could explain why central MG and RGCs are more resistant to HP than the peripheral cells. Among the proteins overexpressed in peripheral MG under exposed to HP as opposed to the control peripheral MG are: ACTN4, a member of the actin binding protein family that interacts with DNaseY and mediates DNA fragmentation during apoptosis [68]; Epha2, from the Eph receptor tyrosine kinase (RTK) family, the largest group of tyrosine kinases in the genome [69], an RTK regulated by p53 proteins that induces apoptosis [70]; FABP3, member of a family of binding proteins that inhibits proliferation and promotes apoptosis when overexpressed [71]; TXN is a key element in the elimination of reactive oxygen species [72]; and PSMA4 that interacts with proteins with a strong immune response [73]. The increase in the proportion of proteins related to these functions suggests that the peripheral MG are more sensitive to HP, and consequently they negatively affect the survival of RGCs and of themselves. In addition, a STRING analysis of several of these proteins show that they interact with proteins also present in the CM of peripheral MG exposed to HP, suggesting that these pathways may be involved in the increased susceptibility of MG to HP and consequently decrease RGC survival.

## Conclusion

**Fig. 8:**
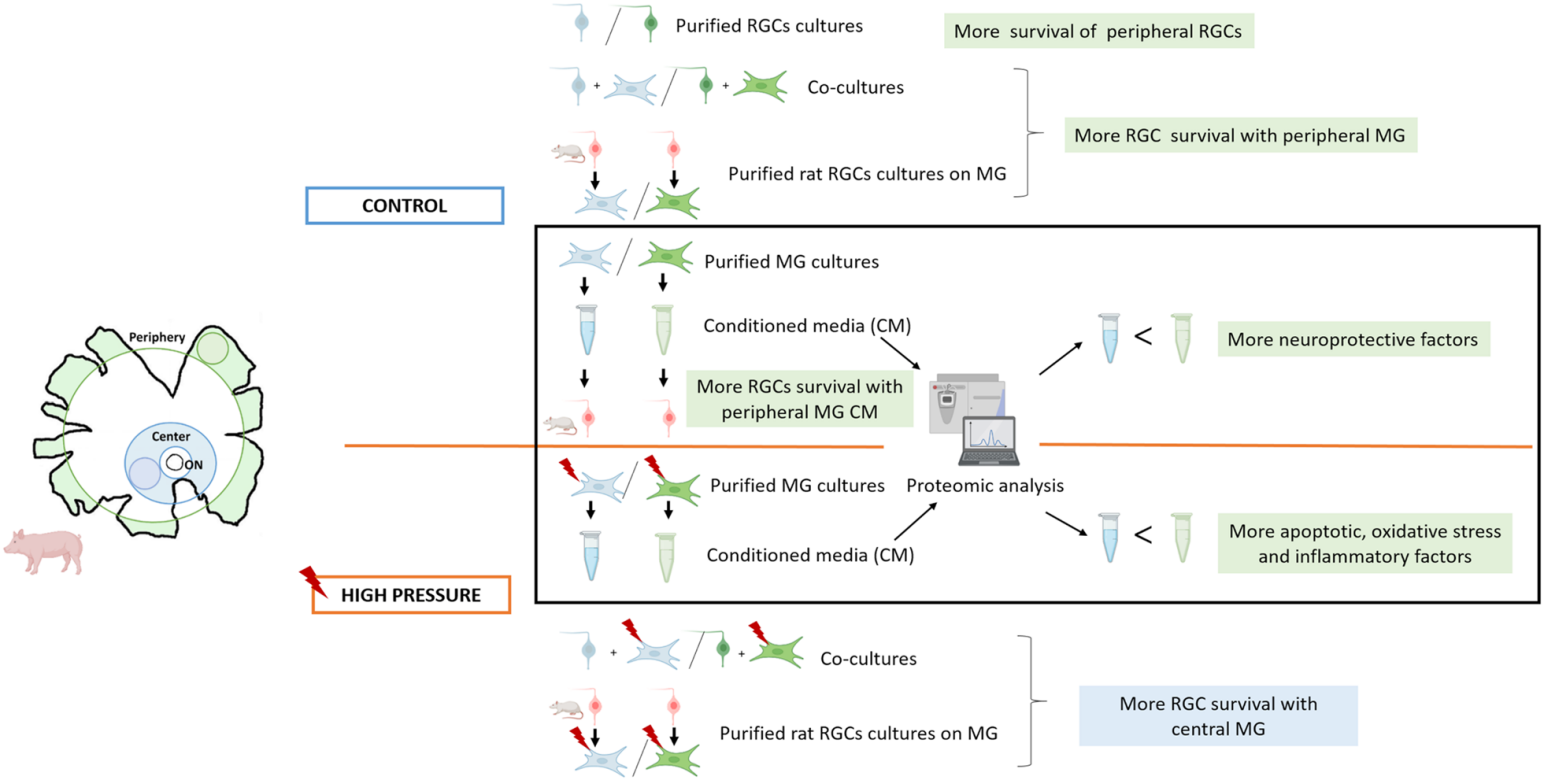
Graphical overview. Visual summary illustrating the key findings and main conclusions of the study

This study (summarized in Fig. 8), demonstrates a clear heterogeneity between MG from the periphery and central retina, based on their behavior and secretion of specific factors in vitro. In control cultures, peripheral MG is more neuroprotective to RGCs, which could be due to being in a more dedifferentiated state. However, peripheral MG are more susceptible to pressure, which causes the secretion of proteins related to apoptosis, oxidative stress and inflammation, which may be implicated in RGC death at the early stages of glaucoma. Better understanding the different factors secreted by subpopulations of MG could identify potential therapeutic targets to enhance retinal neuroprotection and confirm the role of MG in the death of RGCs. We conclude that MG are important sensors of pressure changes in the eye, and they can influence the survival of their neighboring RGCs by secreting different proteins.

### Supplementary Information

**Additional file 1:** Complete proteomic analysis and comparisons of Müller glia Conditioned Media (CM).

## Data Availability

All datasets generated are included in this article.

## Abbreviations

RGCs: Retinal ganglion cells
MG: Müller glia
HP: High pressure
IOP: Intraocular pressure
FBS: Fetal bovine serum
NBA: Neurobasal A
CM: Conditioned medium
PBS: Phosphate buffered saline
BSA: Bovine serum albumin
DAPI: 4′,6-Diamidino-2-phenylindole
αSMA: α-Smooth muscle actin
OCT4: Octamer-binding transcription factor 4
p75NTR: P75 neurotrophin receptor
TRPV4: Transient receptor potential cation channel subfamily V member 4
FASP: Filter aided sample preparation
DTT: DL-Dithiothreitol
FA: Formaldehyde
FDR: False detection rate
STRING: Search tool for the retrieval of interacting genes/proteins
SEM: Standard error of mean
DIV: Days in vitro
DEP: Differentially expressed protein
DKK: Dickkopf
RTK: Receptor tyrosine kinase
